# Molecular Mechanisms of Cold Stress Response in Strawberry and Breeding Strategies

**DOI:** 10.3390/cimb47110966

**Published:** 2025-11-20

**Authors:** Xiang Zhang, Jiajie Yu, Shuang Wang, Rongjia Qiao, Jianjun Shen, Weixiao Li, Fei Zhou, Xiaohong Li

**Affiliations:** School of Agriculture, Liaodong University, Dandong 118003, China; zhangxiang@liaodongu.edu.cn (X.Z.); yujiajie@liaodongu.edu.cn (J.Y.); ws15542199225@163.com (S.W.); 15040251452@163.com (R.Q.); 15140680290@163.com (J.S.); 13644123542@163.com (W.L.); zf_hort2028@163.com (F.Z.)

**Keywords:** cold stress, strawberry, molecular pathway, reactive oxygen species (ROS), antioxidants, cold acclimation, transcription factors, cold-tolerant breeding

## Abstract

As a globally popular crop, strawberry is highly susceptible to cold stress, which significantly limits its cultivation and yield. This review synthesizes current knowledge on the morphological, physiological, and molecular responses of strawberry plants to cold stress. Morphologically, cold stress induces chlorosis, necrosis, and growth retardation, while physiologically, it impairs photosynthesis and membrane integrity and triggers oxidative stress. At the molecular level, the cold acclimation process in plants is orchestrated by a sophisticated regulatory network centered on the ICE-CBF/DREB signaling pathway and incorporating transcription factors, epigenetic modifications, and non-coding RNAs. The accumulation of protective compounds like proline, anthocyanins, and antioxidants is a key metabolic adaptation. Finally, we discuss integrative management practices and future breeding strategies, including genetic engineering, marker-assisted selection, and the use of plant growth-promoting rhizobacteria to enhance cold tolerance. This comprehensive overview provides valuable insights for developing resilient strawberry varieties in the face of unpredictable climate events.

## 1. Introduction

In the context of plant biology, cold stress is a physiological condition triggered by sub-optimal temperatures that disrupt cellular homeostasis, leading to metabolic dysfunction and impaired growth, development, and productivity. Given that only one-third of Earth’s ice-free land is regularly exposed to temperatures below −20 °C, cold stress represents a major and persistent challenge to plant survival [1]. Despite global warming, climate change is driving a trend of more frequent and severe unpredicted low-temperature events. For instance, a single polar vortex event in Texas (USA) in 2021, which caused temperatures to plummet to −18 °C, resulted in catastrophic losses for strawberry growers [2]. In December 2023, record-breaking cold in China’s primary growing regions could delay harvests, reduce fruit quality, and disrupt a multi-billion dollar supply chain [3]. These extreme cold events have caused severe economic, ecologic and societal damages. For instance, the economic repercussions are significant, as demonstrated by specific events: a 2013 frost in China’s Sichuan province caused an estimated ¥7 billion in losses to the local strawberry industry and spurred a 60% surge in purchase prices [4]. Strawberry, known as the “Queen of Fruits”, is one of the seven most popular fruits in the world. Its consistent appeal across diverse markets and consumer groups is driven by a distinctive flavor profile and rich content of nutrients, such as calcium, carbohydrate, dietary fiber, vitamin, trace element, etc. Strawberry is widely planted across the globe, especially in the range between north latitude 30–50° in the Northern Hemisphere. The optimal temperature range for growing strawberry plants is between 10 and 26 °C [5]. Therefore, cold stress poses increasing threats to the strawberry industry.

**Figure 1:**
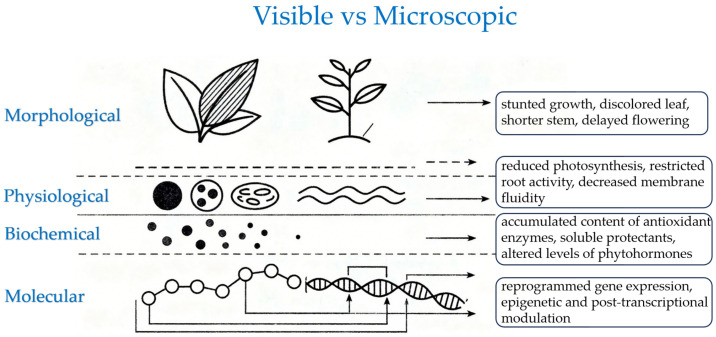
Cold stress triggers multi-level stress responses in plants. Schematic overview of the key changes observed at the morphological (e.g., chlorosis, necrosis), physiological (e.g., impaired photosynthesis, membrane rigidification), biochemical (e.g., ROS accumulation, osmolyte production), and molecular (e.g., ICE-CBF pathway activation) levels. This integrated response highlights the interconnected nature of cold damage and acclimation, where molecular signals drive physiological and visible phenotypic outcomes.

Cold stress in strawberries is defined by sub-optimal temperatures that trigger a suite of coordinated responses, including morphological, physiological, biochemical, and molecular adaptations (Figure 1) [6,7,8]. The manifestation of cold damage in strawberry plants below their optimal temperature range varies significantly, depending on the type of cold stress and the genetic susceptibility of the cultivar. Cold stress can be categorized as chilling stress (0–10 °C) and freezing stress (<0 °C) [9]. Chilling stress influences the stability and activity of proteins and thereby causes membrane rigidification [10,11]. It inhibits essential metabolic processes in strawberry plants like photosynthesis, which leads to stunted leaves, shorter stems, delayed flowering, etc. [12,13,14,15]. However, the severity of these responses exhibits significant genotype-specific variability, a key rationale for targeted breeding programs. The extent of this damage varies; for instance, the widely planted “Camarosa” cultivar is highly sensitive to chilling, showing severe photosynthetic inhibition, whereas “Festival” demonstrates greater resilience with less pronounced growth retardation [16]. Chilling stress also causes leaf discoloration (purple/red pigmentation in leaves) resulting from the accumulation of anthocyanins [17]. Cold soil inhibits the growth and development of strawberry roots and hence reduces nutrient uptake and causes wilting [18]. Compared to other strawberry cultivars, “Benihoppe” shows a relatively robust root system under suboptimal temperatures [19]. Disrupted cell division during flowering can lead to poor pollination and subsequent fruit malformation in strawberries [20]. Freezing stress inflicts severe damage by inducing the formation of intracellular and extracellular ice crystals, which subsequently cause cellular dehydration [21]. They can finally rupture cell membranes and lead to water-soaked, necrotic (black/brown) leaves and flowers [22]. Prolonged freezing stress destroys cell structures and even causes demise of the entire plants [23]. To survive freezing stress, cold-acclimated strawberry plants enter dormancy to conserve energy reserves. [24]. Consequently, delayed flowering and fruiting directly compromise strawberry yield.

Cold stress also causes physiological changes in strawberry plants. It degrades chlorophyll and disrupts the function of chloroplasts, which reduces photosynthetic efficiency [25]. Cold-induced stomatal closure limits CO_2_ uptake and transpiration, which influences a series of metabolic processes [26]. Cold soil restricts root activity and thus affects water and nutrient uptake, leading to drought-like symptoms and nutrient deficiencies [27]. Cold stress also leads to membrane rigidity. Decreased membrane fluidity causes leaks upon rewarming, which disrupts ion homeostasis [12]. At the biochemical level, plants accumulate antioxidant enzymes such as superoxide dismutase (SOD), catalase (CAT), and peroxidase (POD) to scavenge reactive oxygen species (ROS) and mitigate cold-induced oxidative damage [28]. Osmoprotectants, such as soluble sugars (e.g., glucose, raffinose), proteins, and proline, stabilize cellular structures by depressing the intracellular freezing point [29,30,31,32]. Cold stress modulates the levels of key phytohormones, such as abscisic acid and gibberellins, which in turn orchestrate the plant’s transcriptional and physiological responses to low temperatures [33,34,35]. The molecular responses of strawberry plants to cold stress primarily involve transcriptional reprogramming and epigenetic modification. One of the core modules of the gene regulation networks upon cold stress is CBF/DREB (C-repeat binding factor/dehydration-responsive element-binding) transcription factor, which has been extensively studied in various plant species [36,37,38,39,40,41]. For example, functional characterization in the diploid *Fragaria vesca* [42] and the octoploid cultivar confirms its central role [43]. Rapidly induced by cold exposure, CBF transcription factors function by binding to the CRT/DRE (C-repeat/dehydration-responsive element) in the promoters of *COR* (cold-responsive) genes, which are directly related to the cold acclimation of plants [44]. Additionally, plants respond to cold stress in CBF-independent pathways. The genes involved coordinately regulate the response of strawberry plants upon cold stress.

Research on strawberry cold tolerance is increasingly focused on elucidating molecular responses, deciphering gene regulatory networks, and breeding resilient cultivars. This review aims to synthesize current knowledge on strawberry cold stress responses, with a scope encompassing the integrated cascade of events from the initial molecular perception of cold stress to the resulting morphological and physiological symptoms and culminating in the latest applied strategies for enhancing cold tolerance of strawberry plants. It provides a framework for understanding strawberry acclimation mechanisms and opens new avenues for improving cultivation practices.

### Literature Search Methodology

This review synthesizes the current understanding of cold stress responses in strawberry, from molecular mechanisms to breeding applications. To ensure a comprehensive and unbiased survey, the literature was identified through searches in the PubMed, Web of Science, and CNKI (China National Knowledge Infrastructure) databases. Core search queries included combinations of: (“Fragaria” OR “strawberry”) AND (“cold stress” OR “chilling” OR “freezing”) AND (“molecular mechanism” OR “CBF” OR “DREB” OR “antioxidant” OR “breeding”). The search prioritized publications from 2005 to 2025, ensuring the inclusion of modern omics studies while also referencing key foundational papers. The final selection of literature was guided by the criteria of experimental rigor, relevance to the central themes of the review, and the contribution of novel insights.

## 2. Effects of Cold Stress on the Morphological Traits of Strawberry Plants

Cold stress induces a series of distinct morphological changes in strawberry plants. These changes provide a visible record of physiological distress. The symptoms progress predictably across both vegetative and reproductive structures. Initial symptoms are most apparent in foliage. Exposure to chilling conditions typically induces interveinal chlorosis, characterized by yellowing of the leaf tissue while the veins remain green [14,45]. This chlorophyll loss is a direct morphological signal of photosynthetic breakdown and disrupted nutrient assimilation [46]. Jiang et al. find that under a treatment of 10/0 °C for 9 days, the total chlorophyll content (Chl a + b) in strawberry leaves dropped to 73.74% of the level in the control group [47]. Without mitigation, chlorosis can escalate to irreversible necrosis, which manifests as a scorching or browning [48]. It begins at the most vulnerable parts of leaf—the margins and tips. Advancing inward, the progression of this damage causes entire leaves to become desiccated, brittle, and necrotic.

Another overarching symptom is profound growth retardation [14,48]. The entire plant architecture assumes a stunted and dwarfed appearance. This stunting is a direct morphological consequence of suppressed meristematic activity, which can be quantified by the significant variation in cold tolerance between genotypes, with LT50 (the temperature at which 50% of plants die) ranging from −7.7 °C to −12.0 °C in diploid strawberries [49]. Cold stress drastically slows cell division and inhibits the elongation of new cells [50]. It results in a weak, shallow root architecture that cannot effectively anchor the plant or access water and nutrients, which creates a negative feedback loop that worsens the plant decline. A key sign of advanced chilling injury is the phenomenon of water-soaking [51]. Physiologically, this is a direct result of plasma membrane and tonoplast rupture caused by cold-induced rigidification. The loss of membrane semi-permeability leads to massive electrolyte leakage and the efflux of cell fluids into the intercellular spaces (apoplast). This occurs at the cellular level when the integrity of plasmalemma and tonoplast membranes is compromised by rigidifying cold. The loss of semi-permeability causes cell fluids to leak into intercellular spaces, which fundamentally alters tissue structure and leading to cell death [52].

The most economically devastating morphological alterations, however, occur in the reproductive organs. The delicate floral tissues are exquisitely sensitive to freezing temperatures. A direct freeze event causes freeze-induced necrosis, where flowers and developing fruits turn black and collapse entirely [53]. It is a clear and total ablation of reproductive potential. Even non-freezing chilling can cause severe fruit malformation. Cold stress during bloom critically impairs the development and function of male and female gametes, which leads to poor pollination and fertilization [20]. Critically, successful fertilization is a key trigger for the synthesis and redistribution of hormones like auxin and gibberellins (GAs), which orchestrate uniform cell division and expansion in the strawberry receptacle. Cold stress disrupts this hormonal signaling, leading to inadequate and asymmetrical auxin and GA gradients. Morphologically, it leads to poor pollination and fertilization, which disrupts the hormonal signals required for uniform receptacle development. The outcome is a high incidence of asymmetrical, small, and seedy fruits known as “cat-facing” or “button berries”. These malformations are not simply cosmetic flaws; they represent a fundamental failure of fruit set and render the crop entirely unmarketable.

## 3. Effects of Cold Stress on the Physiological Traits of Strawberry Plants

Cold stress profoundly disrupts the core physiological processes of strawberry plants, with the impact most acutely felt through the inhibition of photosynthesis. Research from controlled environment studies shows that chilling temperature impairs the photosynthetic apparatus by damaging chloroplast ultrastructure and inactivating key Calvin cycle enzymes, such as Rubisco [54]. It leads to a significant decline in net photosynthetic rate. Furthermore, while light-harvesting complexes continue to absorb light energy, downstream metabolic inhibition prevents its use in photochemistry, resulting in excess excitation energy. This imbalance creates an energy overload, which results in photoinhibition and the excessive generation of reactive oxygen species (ROS), including superoxide radicals (O_2_^−^) and hydrogen peroxide (H_2_O_2_) [55]. This oxidative burst is a primary driver of cellular damage, as ROS molecules peroxidize lipids in the cellular membranes degrade proteins and disrupt nucleic acids. The integrity of cellular membranes is a critical physiological casualty of cold stress [56]. Membrane fluidity decreases with the drop of temperature, which causes the lipid bilayer to transition from a flexible liquid-crystalline state to a rigid gel-like state. The loss of fluidity compromises membrane permeability and leads to the leakage of electrolytes and essential solutes, which is a metric measured as ion leakage (measured by ion leakage (EC value)) to quantify cold injury [12]. Concomitantly, cold stress disrupts water relations within plants. Reduced root hydraulic conductivity and diminished water uptake would induce a physiological drought even in moist soils. It can cause a drop in leaf water potential and turgor pressure, which contributes to wilting. The regulation of stomatal aperture is a critical, dynamic process in this context. The initial response to cold stress is often a rapid stomatal closure, primarily mediated by a burst of abscisic acid (ABA). This closure reduces transpirational water loss, helping to maintain hydraulic integrity. However, prolonged closure severely limits CO_2_ influx, exacerbating the energy imbalance within the chloroplast and promoting ROS generation. During cold acclimation, a partial and strategic stomatal re-opening can occur. This is thought to be a recovery mechanism to facilitate essential gas exchange and mitigate photoinhibition, likely involving a complex re-regulation of ABA catabolism and sensitivity in guard cells. In response to these challenges, strawberry plants activate complex acclimation mechanisms. This includes the upregulation of antioxidant enzymes like superoxide dismutase (SOD), catalase (CAT), and peroxidase (POD) to scavenge ROS [57]. There is also an accumulation of compatible osmolytes, such as proline and soluble sugars, which act as cryoprotectants by stabilizing proteins and membranes, lowering the intracellular freezing point and mitigating osmotic stress [58]. Ultimately, the physiological toll of cold stress manifests as a massive energetic deficit; the plant must divert energy from growth and development to fuel costly survival and repair mechanisms, which leads to stunted morphology and reduced yields observed in the field.

## 4. Effects of Cold Stress on the Metabolic Traits of Strawberry Plants

Cold stress triggers a distinct reprogramming of primary metabolism in strawberry plants, which focuses on the accumulation of key osmolytes that serve a cryoprotective function. A primary response is the rapid biosynthesis and accumulation of compatible solutes, including amino acids like proline and specific sugars. Proline acts as a molecular chaperone to stabilize proteins and membranes while also functioning as a potent hydroxyl radical scavenger to mitigate oxidative damage [59]. For instance, in the “Paros” cultivar, proline content surged by 239.2% under cold stress [60]. Similarly, raffinose, a trisaccharide, accumulates in chloroplasts and other cellular compartments. It helps to stabilize membrane bilayers and prevent their crystallization during freezing stress, which is crucial for maintaining cell turgor and preventing dehydration injury caused by extracellular ice formation [23,61,62]. Furthermore, there is a notable increase in the levels of other soluble sugars, such as sucrose and fructose, which provide both immediate energy reserves and additional cryoprotection [63]. This reallocation of carbon resources comes at a cost, often diverting energy from growth-related processes, but is essential for short-term survival.

Concurrently, cold stress stimulates the phenylpropanoid pathway, leading to a significant enhancement in the production of secondary metabolites. There is a marked increase in the synthesis of phenolic compounds, particularly anthocyanins, which are responsible for the characteristic deep red pigmentation often observed in cold-acclimated strawberry leaves and fruits [64]. This is not merely a visual symptom; anthocyanins are powerful antioxidants that help quench the reactive oxygen species (ROS) generated by photosynthetic imbalance under low temperatures [65]. Key enzymes in this pathway, such as chalcone synthase (CHS), are directly upregulated by the core cold-signaling network [66]. However, the correlation between cold stress and anthocyanin accumulation is not universal and can be tissue-specific. A pivotal study by Mao et al. (2022) [66] demonstrated that in strawberry fruit, low temperature actively inhibits anthocyanin accumulation, contrary to what is often seen in leaves. The mechanism involves the activation of FvMAPK3, which phosphorylates the key transcription factor FvMYB10 and promotes the degradation of Chalcone Synthase 1 (FvCHS1), a critical enzyme in the anthocyanin pathway [58]. This suppression of pigmentation in fruits results in the commercially undesirable ‘paling’ of strawberries in cold storage. Therefore, while anthocyanin accumulation in vegetative tissues is often a hallmark of cold acclimation and contributes to tolerance by mitigating oxidative stress, its specific role and regulation depend on the organ and the complex interplay of the underlying signaling pathways [66]. Studies have shown that strawberries grown under moderate cold stress exhibited fluctuated levels of various flavonoids like quercetin and kaempferol derivatives [67]. The hormonal landscape undergoes a profound shift to orchestrate the metabolic and physiological changes required for cold acclimation, characterized by extensive cross-talk between signaling pathways. While abscisic acid (ABA) is a well-established master regulator of cold responses, other hormones like brassinosteroids (BRs) and ethylene play critical modulating roles. For example, the application of 24-epibrassinolide (EBL), a brassinosteroid, has been found to further enhance the cold-induced accumulation of protectants while helping to maintain membrane integrity [68].

Brassinosteroids and ABA often exhibit a synergistic relationship under cold stress [69]. EBL application can enhance the plant sensitivity to ABA, leading to a stronger activation of antioxidant defense systems and a more robust induction of key cold-responsive genes, including those in the CBF regulon [68]. Conversely, ABA can also influence BR signaling, creating a positive feedback loop that amplifies the stress response. In contrast, the relationship between BRs and ethylene can be more antagonistic. Cold stress often induces ethylene production, which can promote leaf senescence and chlorophyll degradation. BRs, however, can act to suppress ethylene biosynthesis or signaling, thereby mitigating its senescence-promoting effects and helping to preserve photosynthetic tissues under prolonged cold stress. This dynamic cross-talk between BRs, ABA, and ethylene ensures a finely balanced response: ABA and BRs cooperate to activate protective mechanisms, while BRs counterbalance the potentially detrimental effects of ethylene, collectively promoting survival without prematurely committing to senescence.

## 5. Molecular Pathways in Strawberry Plants upon Cold Stress

### 5.1. Initial Cold Sensing and Signal Transduction

The molecular response of strawberry plants to cold stress is initiated by sophisticated sensing and signal transduction mechanisms. Key primary events include rapid calcium ion (Ca^2+^) fluxes, occurring within seconds to minutes of stress onset, and the controlled generation of reactive oxygen species (ROS) by NADPH oxidases, such as FvRbohA and FvRbohD [70,71]. These signals are propagated through phosphorylation cascades, notably the mitogen-activated protein kinase (MAPK) pathways. This initial signaling network activates the core transcriptional machinery responsible for cold acclimation, which sets the stage for a complex gene regulatory response under suboptimal temperatures. Furthermore, MAPK signaling pathway plays a significant role in transmitting cold signals in strawberries. Research has found that under cold stress, FvMAPK3 in strawberry was activated, which acted as a downstream target [66]. It was phosphorylated by MAPK KINASE4 (FvMKK4) and SUCROSE NONFERMENTING1-RELATED KINASE2.6 (FvSnRK2.6), which in turn phosphorylated the transcription factor FvMYB10. These findings are supported by protein-level evidence, moving beyond transcriptomic data. MAPK pathways act as a key regulatory module that amplifies and specifies the cold stress signal to activate the CBF-dependent transcriptional cascade. This cascade regulates the expression of related genes and affects anthocyanin accumulation.

### 5.2. Core Transcriptional Regulatory Network

Research into the genetic basis of low-temperature stress in strawberry deepens our understanding of its cold adaptation mechanisms. In woodland strawberry (*Fragaria vesca*), 37 NAC transcription factor genes displayed tissue-specific expression patterns [72]. The expression of numerous genes was significantly altered by diverse abiotic stresses—such as low temperature, high temperature, drought, and salt—as well as by phytoregulator treatments. It indicated that these genes play important roles in the response of strawberry to multiple stresses, including low-temperature stress. Furthermore, studies on strawberry flowering time have demonstrated that its phenotypic plasticity is modulated by the interaction between genetic loci and ambient temperature [73]. By analyzing a biparental segregating population planted at different latitudes and combining climate variables with genomic data, 25 quantitative trait loci (QTL) associated with flowering time are detected. The effect of some of these QTLs, such as the 6D_M QTL, is strongly modulated by temperature. It provides important clues for genetically improving the adaptability of strawberry to different environmental temperatures.

The core of regulatory network are key transcription factors that act as master switches. The C-repeat binding factor (CBF)/Dehydration Responsive Element Binding (DREB) pathway is a central module. Modern multi-omics approaches have been instrumental in moving beyond correlative observations to establishing causal mechanisms in strawberry cold tolerance. A prime example is the elucidation of the role of FveDREB1B in the diploid woodland strawberry (*Fragaria vesca*). In a transcriptomic analysis of cold-stressed strawberries, FveDREB1B is identified as one of the most strongly induced transcription factors. Concurrent metabolomic profiling revealed a significant cold-induced accumulation of specific protective compounds, including raffinose (an osmoprotectant) and anthocyanins (antioxidants). The results of ChIP-seq revealed that FveDREB1B directly binds to the promoters of key biosynthetic genes, including *FveSCL23* and *FveCHS*. [42]. While the FveDREB1B in diploid *F. vesca* function by directly regulating downstream genes, the octoploid *FaTINY2* appears to function through a broader enhancement of antioxidant capacity. The overexpression of this gene in *Arabidopsis* reduces the accumulation of reactive oxygen species (ROS) and malondialdehyde (MDA) under low-temperature and salt stress [43]. This functional divergence raises intriguing questions about gene duplication and sub-functionalization in polyploid strawberries. The levels of proline and chlorophyll, as well as the activities of CAT, SOD, and POD enzymes, exhibit increased levels, which indicates that FaTINY2 enhances plant stress tolerance by regulating related genes. Otherwise, the stability of upstream regulators is crucial. The *FvICE1* gene isolated from strawberry, which belongs to the bHLH transcription factor family, shows significantly upregulated expression in different strawberry tissues after treatments such as low temperature, drought, salt, and heat [74]. Strawberries overexpressing *FvICE1* exhibit enhanced tolerance to low temperature and drought, whereas *fvice1* mutant strawberries obtained via CRISPR/Cas9 technology show reduced tolerance. The expression of related cold-responsive genes such as *FvCBF1* and *FvCBF2* is also positively regulated or suppressed, which reveals the key role of *FvICE1* in strawberry plants response to low temperature and drought stress. These studies suggest that while the core ICE-CBF module is universal, its downstream targets and regulatory fine-tuning may vary significantly between diploid and octoploid species, a critical consideration for cross-species applications in breeding.

### 5.3. Epigenetic and Post-Transcriptional Modulation

Beyond transcription factors, epigenetic regulation and chromatin remodeling govern a critical layer of gene control. A key discovery involves the FvMSI4/FVE protein, which acts as a scaffold to recruit a repressive complex containing the histone deacetylase FvHDA6 and the E3 ligase FvHOS1 [6]. This complex likely modulates gene expression by altering histone acetylation marks, which repress genes that inhibit cold tolerance, such as those promoting flowering. These mechanisms find a powerful parallel in the well-established phenomenon of vernalization in *Arabidopsis thaliana*, which represents a classic example of stable ‘cold memory’. Prolonged winter cold induces the epigenetic silencing of the *FLC* gene, a potent flowering repressor. This is achieved through the recruitment of Polycomb group proteins to a specific cold memory element, which catalyze the deposition of the repressive H3K27me3 mark, forming a stable silent chromatin domain that is maintained long after temperatures rise [75]. This allows the plant to “remember” its winter experience and flower in spring. In another *Arabidopsis* pathway, cold stress triggers the HOS15-mediated degradation of the histone deacetylase HD2C, switching the chromatin of COR genes from a repressive to a permissive state to activate cold tolerance [76]. Furthermore, studies in the wild potato species *Solanum commersonii* indicate that key cold signaling pathways, such as the MAPK pathway, which can interact with epigenetic regulators, are also upregulated under freezing stress [77]. This demonstrates the evolutionary conservation of chromatin-level regulation in plant cold response. Non-coding RNAs also play an important role in the strawberry plants cold stress response. Research shows that non-coding RNAs such as long non-coding RNAs (lncRNAs) and microRNAs (miRNAs) are involved in regulating the strawberry plants response to low temperature stress. For example, a study on the strawberry fruit ripening process proves that a lncRNA, FRILAIR, acts as a non-classical target mimic of miR397 to regulate the expression of *LAC11a*, thereby influencing the fruit ripening process [78]. It suggests a regulatory role for non-coding RNAs in strawberry growth, development, and stress response. Furthermore, under low temperature stress, some miRNAs in strawberry exhibit fluctuation in expression, which in turn regulate the expression of their target genes. Research has shown that miR164 modulates senescence in cold-stored strawberry fruit by negatively regulating NAC transcription factors [79]. This creates a coordinated response: while the CBF network activates stress-protective genes, parallel mechanisms like those mediated by FvMSI4/FVE and miR164 simultaneously repress developmental programs that are energetically costly or incompatible with cold survival.

### 5.4. Downstream Metabolic Reprogramming and Integrated Network

The integrated understanding of this cold stress network provides a robust molecular framework for Marker-Assisted Selection (MAS) to accelerate the breeding of cold-tolerant strawberries. Key components of this network serve as ideal targets for MAS. The activation of transcription factors like CBFs, ERFs, and ICE1 leads to the expression of a suite of protective effector genes. This includes the synthesis of dehydrins (e.g., Xero2) for cellular protection and the accumulation of compatible solutes like proline and raffinose for osmoregulation [42,68,80]. The synthesis of raffinose is directly regulated by cold-induced transcription factors. The direct targeting of *FveCHS* by FveDREB1B also promotes the biosynthesis of protective secondary metabolites, including anthocyanins and phenolic compounds like chlorogenic acid, which contributes to the characteristic reddening of cold-stressed tissues and provided potent antioxidant activity. By using molecular markers linked to these genes, breeders can efficiently practice gene pyramiding and early-generation selection to develop new cultivars with a robust and coordinated cold tolerance mechanism, bypassing the need for laborious and less reliable phenotypic screening alone.

**Figure 2:**
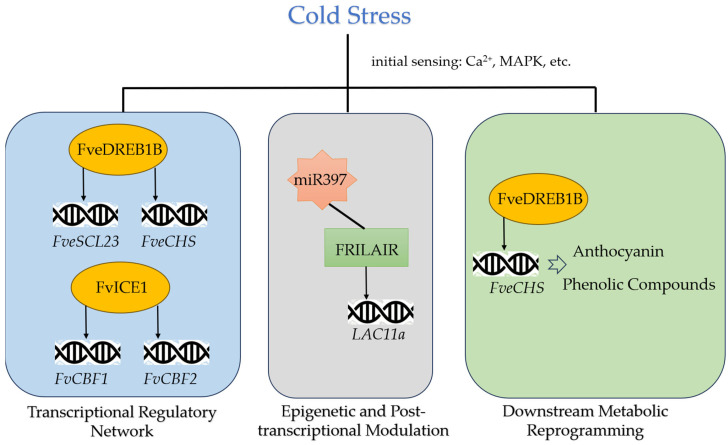
A proposed integrated network for molecular cold stress signaling in strawberry. The model illustrates how initial cold sensing (Ca^2+^ flux, ROS burst) activates a core transcriptional cascade centered on the ICE-CBF/DREB module. This core is fine-tuned by epigenetic regulation (e.g., FvMSI4/FVE complex) and post-transcriptional control by non-coding RNAs (miRNAs, lncRNAs), which collectively orchestrate downstream metabolic reprogramming (e.g., raffinose and anthocyanin synthesis). Solid arrows represent direct transcriptional activation based on cited evidence; swallow-tailed arrows represent regulatory adjustments. This network depicts a highly coordinated mechanism for achieving cold acclimation.

Substantially, the molecular response to cold in strawberry is a highly integrated network, rather than a linear pathway (Table 1, Figure 2). It seamlessly combines initial calcium/ROS signaling, core transcriptional cascades (ICE-CBF-DREB), epigenetic control via histone modification, and sophisticated post-transcriptional regulation by non-coding RNAs. This network precisely coordinates downstream physiological and biochemical responses to mitigate cold-induced damage.

## 6. Discussion

This review systematically illustrated the multi-layered response of strawberry plants to cold stress and presented a continuum of adaptation from the macroscopic to the molecular level (Table S1). The evidence consolidates that cold stress is not a singular injury but a complex syndrome that manifests through interconnected morphological, physiological, biochemical, and molecular alterations.

Morphologically, the visible decline of plants—through chlorosis, necrosis, stunted growth, and reproductive failure—is the ultimate expression of internal physiological dysfunction. At the physiological level, the inhibition of photosynthesis and the breakdown of membrane integrity are pivotal events, leading to an energy crisis and oxidative stress. In response, strawberry plants activate a sophisticated biochemical defense system, characterized by the accumulation of compatible osmolytes like proline and raffinose for osmotic adjustment and the synthesis of antioxidant compounds like anthocyanins to quench reactive oxygen species (ROS). Underpinning these changes is a highly regulated molecular network. The ICE-CBF/DREB transcriptional cascade acts as a master switch, coordinating the expression of protective genes. The activation of this pathway leads directly to the synthesis of COR (Cold-Regulated) gene products, such as dehydrins and key enzymes for osmolyte biosynthesis. This molecular response confers field resilience by stabilizing cellular structures and maintaining photosynthetic function during cold snaps, which is a direct determinant of final yield maintenance. Simultaneously, to ensure long-term survival, the plant delays reproductively sensitive stages to avoid cold damage. This is achieved through epigenetic mechanisms, such as histone modification mediated by the FvMSI4/FVE complex, which represses genes that promote flowering. This strategic molecular delay in flowering, while potentially extending the time to harvest, is a critical trade-off that safeguards the plant’s reproductive potential, ensuring that flowering and fruit set occur under more favorable conditions and ultimately preventing total crop loss. Our synthesis reveals that the strawberry’s molecular response to cold is not a linear pathway but a tightly integrated network with the ICE-CBF/DREB cascade at its core. However, a critical insight from comparing studies across diploid and octoploid species is the apparent divergence in how this core is utilized. Furthermore, the emerging picture is one of resource allocation trade-offs, elegantly demonstrated by the FvMSI4/FVE complex’s repression of flowering. This suggests a unifying principle: cold tolerance is achieved not only by activating protective mechanisms but also by the active suppression of competing developmental processes. The challenge for future breeding will be to manipulate this network to enhance protection without crippling growth and yield.

The integration of these responses highlights a fundamental trade-off: resources are diverted from growth and development to fuel survival mechanisms. This reallocation ensures short-term survival under stress but comes at the cost of reduced yield and fruit quality. However, the detailed understanding of the underlying cold signaling network now enables precise biotechnological interventions to mitigate this trade-off. Targeted genetic engineering and gene editing (e.g., CRISPR-Cas9) can be deployed to fine-tune this response. For instance, overexpression of master regulators like ICE1 or CBF can be spatially or temporally controlled to enhance the expression of protective genes (e.g., dehydrins, osmolytes) specifically when cold stress is imminent, thereby pre-emptively boosting tolerance. Conversely, genes that strongly inhibit growth under stress could be knocked down to lessen the yield penalty. By strategically manipulating key nodes in the signaling network, the goal is to engineer strawberry cultivars that mount a robust defense without completely sacrificing growth and productivity, ultimately decoupling survival from severe yield loss.

**Figure 3:**
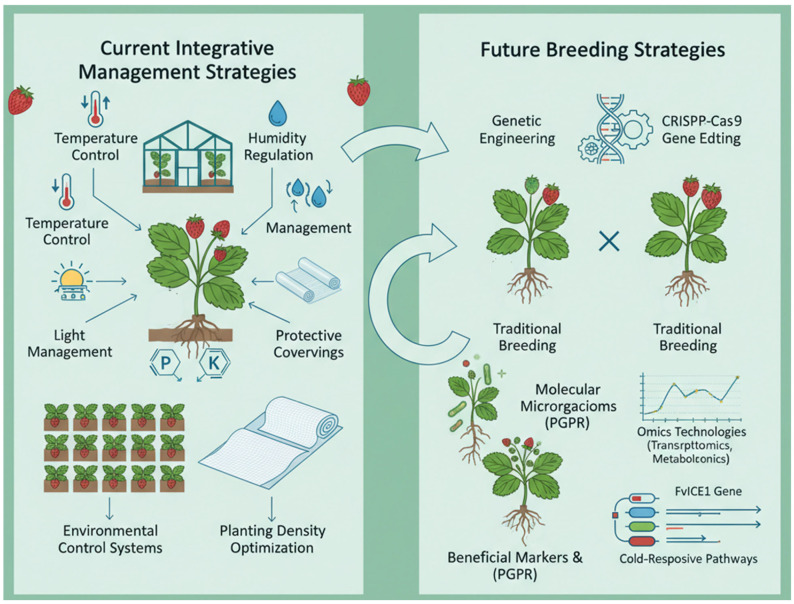
Strategies for enhancing cold tolerance in strawberry span from immediate agronomic practices to long-term genetic solutions. The left side summarizes integrative management practices (e.g., optimized fertilization, protective cultivation) that mitigate cold damage. The right-side outlines future breeding strategies, including genetic engineering (e.g., overexpressing *FvICE1*), marker-assisted selection (MAS), and genome editing (e.g., CRISPR-Cas9), which aim to develop resilient, next-generation cultivars. The synergy between these approaches is essential for safeguarding strawberry production against climate instability.

The profound understanding of these mechanisms, as summarized above, provides a solid foundation for developing strategies to enhance cold tolerance in strawberry. While integrative management practices offer immediate protection, the future of strawberry breeding lies in leveraging this molecular knowledge (Figure 3).

### 6.1. Integrative Management Strategies for Mitigating Cold Stress Damage in Strawberry Plants

Protecting strawberry cultivation from the detrimental effects of cold stress necessitates an integrated approach that combines immediate agronomic interventions with precise environmental control. Current management strategies are fundamentally focused on enhancing the plant inherent resilience and modifying the microclimate to avert exposure to damaging temperatures. A primary component involves refined cultivation practices. In terms of cultivation management, reasonably adjusting planting density and optimizing fertilization and irrigation measures can help improve the cold resistance of strawberry plants. For instance, the application of phosphorus and potassium-rich fertilizers is a well-established practice to bolster plant stress resistance [81]. This nutritional strategy strengthens cell walls and improves osmotic regulation, which contributes to greater membrane stability during chilling events.

Concurrently, precise environmental management is critical, especially within protected cultivation systems. In facility cultivation, regulating environmental factors such as temperature, light, and humidity creates a suitable growth environment for strawberry. It involves a tiered approach to insulation, where secondary layers of film or non-woven fabric are deployed as temperatures approach critical thresholds to minimize radiant heat loss. Concrete research on substrate warming systems demonstrates the significant benefits of targeted root-zone heating. Wei et al.’s research shows that applying this technology increased the average fruit yield per plant by 17.8% and the proportion of premium fruits by 28.6% [82]. Furthermore, managing humidity through controlled ventilation is essential to prevent conditions that favor fungal pathogens without inducing additional chilling stress. In addition, measures such as covering for heat preservation and ventilation can also optimize the micro-environment for strawberry growth, enhancing its adaptability to low temperature. The synergy between these agronomic and environmental strategies—fertilization, irrigation, planting density, and active microclimate control—forms a robust first line of defense against cold stress, helping to maintain productivity and fruit quality under suboptimal conditions.

### 6.2. Future Directions for Breeding Cold-Tolerant Strawberry Plants

Around the world, strawberry is making great efforts to coping with cold adaptation. For instance, research on day-neutral strawberries in Chile suggests a slight southward shift in suitable cultivation areas under warming scenarios. The key to success in these new areas will be implementing strategies specifically to minimize freezing damage to the more vulnerable flowers, highlighting the need for location-specific cultivation plans. The Chinese strawberry industry is transitioning from pursuing high yield to focusing on high quality and the development of derivative products. Breeding and selecting varieties with better cold tolerance is a key part of this shift. The future of enhancing cold tolerance in strawberry lies in a multi-faceted biotechnological framework that integrates advanced genetic engineering, precision breeding, and novel microbial technologies. Tolerance breeding is an important strategy for improving the low-temperature resistance of strawberry. Through genetic engineering means, introducing genes related to low-temperature tolerance into strawberry plants can effectively improve their cold resistance. For example, the *FvICE1* gene from strawberry is introduced into strawberry plants via an overexpression vector [74]. Strawberries overexpressing *FvICE1* show enhanced tolerance to both low temperature and drought, and the expression of related cold-responsive genes such as *FvCBF1* and *FvCBF2* is also positively regulated. It demonstrates the potential of genetic engineering to enhance the core cold-signaling network.

Furthermore, the combination of traditional breeding methods with modern molecular technology is also an important pathway. The breeding of cold-tolerant strawberry varieties can be accelerated by screening for superior cold-hardiness and employing molecular marker-assisted selection (MAS). This integrated approach allows breeders to efficiently select superior genotypes [83]. Biotechnology has broad the application prospects in addressing strawberry cold stress. Genome editing, particularly the CRISPR-Cas9 system, represents a paradigm shift. For example, using gene editing technology such as CRISPR/Cas9, the strawberry genome can be precisely edited to knock out or modify genes associated with cold sensitivity, thereby improving cold hardiness [83]. It has been proven that CRISPR-Cas9 system is well-established and functional in strawberries [84]. In addition, the patent from Nanjing Agricultural University provides a forward-looking example [85]. It shows that research is moving beyond simple gene editing to more sophisticated regulation, such as using CRISPR to control the epigenetic state of key cold-response genes like ICE1. Simultaneously, omics technologies provide a systems-level understanding of the stress response. Through techniques such as transcriptomics and metabolomics, a comprehensive understanding of the gene expression and metabolic changes in strawberry under low-temperature stress provides a basis for mining key cold-tolerance genes and metabolic pathways. This knowledge is essential for identifying the most effective targets for genetic manipulation.

Looking beyond the plant genome, exploiting beneficial plant-microbe interactions offers a promising, sustainable strategy. Research has found that some Plant Growth-Promoting Rhizobacteria (PGPR) can form a symbiotic relationship with strawberry roots, which improves the plant tolerance to low temperature by regulating phytohormone levels and enhancing antioxidant capacity. For instance, under low-temperature stress, certain PGPR strains can promote the activity of antioxidant enzymes within strawberry plants, reduce the accumulation of reactive oxygen species, and thereby alleviate the damage caused by low temperature to strawberries [86]. The application of these microbial technologies provides new pathways and methods for solving the problem of strawberry cold stress.

Simultaneously, advanced phenotyping platforms that fuse hyperspectral and chlorophyll fluorescence imaging with AI models like XGBoost allow for non-invasive, high-throughput screening of breeding populations for cold stress responses [87,88]. Looking ahead, emerging research points to new frontiers: epigenetic regulators like the FvMSI4/FVE-HDA6 complex offer novel targets for fine-tuning the cold acclimation response [6], while genes such as CBF4 and Xero2 present prime candidates for precision breeding via genome editing [80]. Finally, the application of AI extends beyond the field into the supply chain, where predictive models can optimize post-harvest logistics to reduce losses, creating a comprehensive strategy from breeding to market [89,90].

### 6.3. Translational Potential Across Rosaceae and Integrated Breeding Frameworks

The molecular insights and biotechnological tools developed in strawberry have significant translational potential for improving cold tolerance in other economically important Rosaceae members, such as raspberry and blackberry. For instance, the highly efficient, visual CRISPR-Cas9 system (NVSR) developed in diploid strawberry has shown preliminary success in raspberry, demonstrating the transferability of this precision breeding technology within the family [91]. Furthermore, key cold tolerance biomarkers identified in *Fragaria*, including Alcohol Dehydrogenase (ADH), dehydrins, and the metabolite galactinol, are conserved across plant species [49]. These biomarkers provide a foundation for establishing marker-assisted selection (MAS) programs not only in strawberry but also in related Rosaceae crops, enabling breeders to rapidly screen and select for enhanced freezing tolerance. This integrated approach, which connects physiological assessments like freezing LT50 with molecular profiling, is crucial for a multi-scale understanding of cold adaptation.

## 7. Conclusions

Cold stress imposes a significant threat to strawberry cultivation, triggering a cascade of detrimental effects ranging from morphological damage to metabolic disruption. This review synthesized the intricate mechanisms underpinning the response of strawberry plants and highlighted the critical role of the ICE-CBF/DREB regulatory core and its associated network of transcriptional, epigenetic, and post-transcriptional controls. These molecular events orchestrate essential biochemical adaptations, including the accumulation of protective osmolytes and antioxidants.

Although conventional agronomy provides essential mitigation, a new paradigm is emerging through the integration of advanced biotechnologies, including genetic engineering, marker-assisted selection, and genome editing. The development and widespread adoption of cold-tolerant cultivars promise substantial economic benefits, including a direct reduction in annual crop losses from unpredictable frost events, lower costs for protective measures and insurance, and greater yield stability for growers, thereby strengthening the entire supply chain.

Leveraging the growing understanding of strawberry cold tolerance genetics will be paramount for developing resilient, high-yielding varieties, ultimately safeguarding strawberry production against the backdrop of climatic instability. However, a key future challenge lies in effectively integrating high-throughput genomics with advanced phenomics, that is, linking the vast data from DNA sequencing with large-scale, precise measurements of plant performance in the field. Overcoming this hurdle will be crucial for deciphering the complex interactions between genotype and environment and for accelerating the breeding of resilient, high-yielding varieties. Ultimately, this integrated approach is essential for safeguarding strawberry production against the backdrop of climatic instability.

## Figures and Tables

**Table 1:** Key molecular players in strawberry cold stress response and their validated functions.

Gene/Protein	Species	Type	Main Function in Cold Response	Evidence (Overexpression/Knockout)	Interacting Partners/Pathway
*FvICE1*	*F. vesca*	TF (bHLH)	Master regulator; enhances tolerance, positively regulates FvCBF1/2	OE: Tolerance ↑, KO: Tolerance ↓ [74]	Upstream of CBFs
*FveDREB1B*	*F. vesca*	TF (AP2/ERF)	Binds promoters of FveSCL23, FveCHS	OE: Tolerance ↑ [42]	CBF/DREB core
*FaTINY2*	*F. x ananassa*	TF (AP2/ERF)	Enhances antioxidant capacity (SOD, CAT, POD), increases proline	OE in Arabidopsis: Tolerance ↑ [43]	CBF/DREB-related
FvMAPK3	*F. vesca*	Kinase	Phosphorylated by FvMKK4/FvSnRK2.6; phosphorylates FvMYB10	Functional analysis [66]	MAPK signaling
FvMSI4/FVE	*F. vesca*	Scaffold	Recruits FvHDA6/FvHOS1 complex; represses flowering	Functional analysis [6]	Epigenetic repression
miR164	*F. x ananassa*	miRNA	Negatively regulates NAC TFs; delays fruit senescence	Expression analysis [79]	Post-transcriptional

↑ sign means increased. ↓ sign means decreased.

## Data Availability

No new data were created or analyzed in this study. Data sharing is not applicable to this article.

## Supplementary Materials

The following supporting information can be downloaded at: https://www.mdpi.com/article/10.3390/cimb47110966/s1.
