# Supplementary material for: Molecular Mechanisms of Cold Stress Response in Strawberry and Breeding Strategies
Source: Curr Issues Mol Biol. 2025 Nov 20;47(11):966. doi: 10.3390/cimb47110966 (PMC12651795; doi:10.3390/cimb47110966)
Supplement: Supplementary file 1 [file cimb-47-00966-s001.zip › cimb-3927720-supplementary.pdf]

Table S1. Effects of low temperature on the morphological, physiological, biochemical, and molecular responses of strawberry plants

Level of Response	Key Effects of Low Temperature
**Morphological**	**Chlorosis & Necrosis:** Yellowing between leaf veins, advancing to brown, necrotic tissue.<br>**Growth Retardation:** Stunted and dwarfed appearance of shoots and roots.<br>**Water-Soaking:** Translucent, limp patches on leaves due to membrane rupture.<br>**Reproductive Failure:** Blackened/collapsed flowers from freezing; misshapen "cat-faced" fruits from chilling.
**Physiological**	**Photosynthetic Inhibition:** Damage to chloroplasts and Calvin cycle enzymes.<br>**Membrane Rigidification:** Loss of fluidity leading to electrolyte leakage.<br>**Oxidative Stress:** Photoinhibition leads to overproduction of ROS (O_2^-, H_2O_2).<br>**Water & Nutrient Imbalance:** Reduced root conductivity induces physiological drought.
**Biochemical**	**Osmolyte Accumulation:** Biosynthesis of proline, raffinose, and soluble sugars for cryoprotection.<br>**Antioxidant System Activation:** Upregulation of enzymes like SOD, CAT, and POD to scavenge ROS.<br>**Secondary Metabolite Production:** Enhanced synthesis of anthocyanins and phenolic compounds.
**Molecular**	**Signal Transduction:** Rapid Ca^{2+} flux and ROS signaling activate MAPK cascades (e.g., FvMAPK3).<br>**Core Transcriptional Regulation:** ICE-CBF/DREB pathway activation induces *COR* genes (e.g., dehydrins).<br>**Epigenetic & Post-Transcriptional Control:** Chromatin remodeling (e.g., via FvMSI4/FVE) and regulation by miRNAs/lncRNAs (e.g., miR164).
